# 3-[4-(Acetamido)­benzene­sulfonamido]­benzoic acid

**DOI:** 10.1107/S1600536810048397

**Published:** 2010-12-04

**Authors:** Sidra Muzaffar Mirza, Ghulam Mustafa, Islam Ullah Khan, Muhammad Zia-ur-Rehman, Muhammad Shafiq

**Affiliations:** a Department of Chemistry, Government College University, Lahore 54000, Pakistan; b Applied Chemistry Research Centre, PCSIR Laboratories Complex, Lahore 54600, Pakistan

## Abstract

In the title compound, C_15_H_14_N_2_O_5_S, the dihedral angle between the aromatic rings is 63.20 (11) Å. The crystal structure displays classical inter­molecular O—H⋯O hydrogen bonding typical for carb­oxy­lic acids, forming centrosymmetric dimers. These dimers are further connected by N—H⋯O and C—H⋯O hydrogen bonds to form an extended network.

## Related literature

For the synthesis of related compounds, see: Khan *et al.* (2009 ▶); Arshad *et al.* (2008 ▶). For the biological activity of sulfonamides, see: Esteve & Bidal (2002 ▶); Hanson *et al.* (1999 ▶); Lee & Lee (2002 ▶); Moree *et al.* (1991 ▶); Ozbek *et al.* (2007 ▶); Parari *et al.* (2008 ▶); Ratish *et al.* (2009 ▶); Rough *et al.* (1998 ▶); Selnam *et al.* (2001 ▶); Soledade *et al.* (2006 ▶); Xiao & Timberlake (2000 ▶). For related structures, see: Gowda *et al.* (2007*a* ▶,*b* ▶,*c* ▶); Haider *et al.* (2009 ▶). For bond-length data, see: Allen *et al.* (1987 ▶).
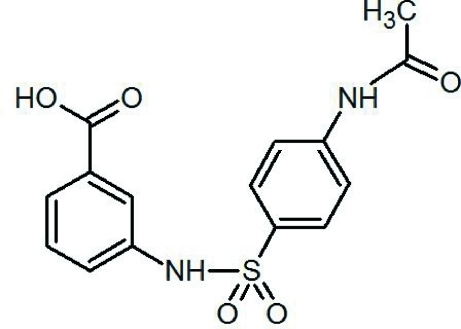


## Experimental

### 

#### Crystal data

C_15_H_14_N_2_O_5_S
*M* *_r_* = 334.34
Triclinic, 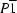

*a* = 7.9829 (3) Å
*b* = 8.4143 (3) Å
*c* = 12.6554 (5) Å
α = 70.888 (2)°
β = 81.553 (2)°
γ = 77.104 (2)°
*V* = 780.44 (5) Å^3^
*Z* = 2
Mo *K*α radiation
μ = 0.23 mm^−1^
*T* = 296 K
0.24 × 0.18 × 0.14 mm

#### Data collection

Bruker APEXII CCD area-detector diffractometer
13620 measured reflections
3835 independent reflections
2928 reflections with *I* > 2σ(*I*)
*R*_int_ = 0.032

#### Refinement

*R*[*F* ^2^ > 2σ(*F* ^2^)] = 0.054
*wR*(*F* ^2^) = 0.150
*S* = 1.02
3835 reflections
210 parameters
H-atom parameters constrained
Δρ_max_ = 0.49 e Å^−3^
Δρ_min_ = −0.47 e Å^−3^

### 

Data collection: *APEX2* (Bruker, 2007 ▶); cell refinement: *SAINT* (Bruker, 2007 ▶); data reduction: *SAINT*; program(s) used to solve structure: *SHELXS97* (Sheldrick, 2008 ▶); program(s) used to refine structure: *SHELXL97* (Sheldrick, 2008 ▶); molecular graphics: *PLATON* (Spek, 2009 ▶) and *Mercury* (Macrae *et al.*, 2006 ▶); software used to prepare material for publication: *PLATON*.

## Supplementary Material

Crystal structure: contains datablocks I, global. DOI: 10.1107/S1600536810048397/sj5052sup1.cif

Structure factors: contains datablocks I. DOI: 10.1107/S1600536810048397/sj5052Isup2.hkl

Additional supplementary materials:  crystallographic information; 3D view; checkCIF report

## Figures and Tables

**Table 1:** Hydrogen-bond geometry (Å, °)

*D*—H⋯*A*	*D*—H	H⋯*A*	*D*⋯*A*	*D*—H⋯*A*
O5—H7⋯O4^i^	0.88	1.74	2.617 (4)	170
N1—H1⋯O16^ii^	0.86	2.31	2.860 (2)	122
N3—H3⋯O1^iii^	0.86	2.13	2.974 (2)	165
C11—H11⋯O2^iv^	0.93	2.59	3.379 (3)	143

Symmetry codes: (i) 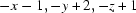; (ii) 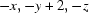; (iii) 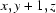; (iv) 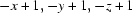.

**Fig. 1.**
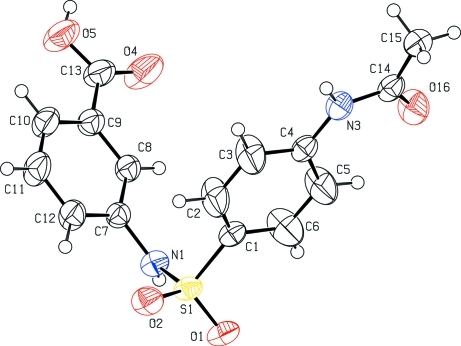
The molecular structure of (I), with displacement ellipsoids drawn at the 50% probability level.

**Fig. 2.**
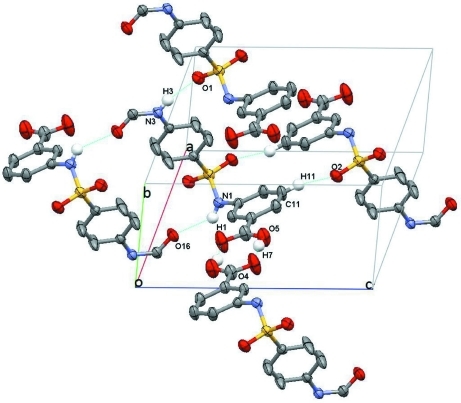
Perspective view of the three-dimensional crystal packing showing hydrogen-bonded interactions (dashed lines). H atoms not involved in hydrogen bonding have been omitted for clarity.

## supplementary crystallographic information

### Comment

Sulfonamides are well known in literature for their potential as biologically active compounds (Hanson *et al.*, 1999; Moree *et al.*,1991; Rough *et al.*, 1998). These have been reported to display anti-hypertensive, anti-convulsant, herbicidal and anti-malarial activities (Esteve & Bidal, 2002; Soledade *et al.*, 2006; Xiao & Timberlake, 2000; Lee & Lee, 2002). In addition the sulfonamide unit has been found in a number of compounds possessing anti-HIV (Selnam *et al.*,2001), anti-inflammatory (Ratish *et al.*, 2009) and anti-microbial (Ozbek *et al.*, 2007; Parari *et al.*, 2008) activities.

In continuation of our work regarding the synthesis of various sulfur containing heterocycles (Arshad *et al.*, 2008; Khan *et al.*, 2009), the structure of 3-({[4-(acetylamino)phenyl]sulfonyl}amino)benzoic acid (I) has been determined. Bond lengths and bond angles of the title molecule (Fig 1) are similar to those in related compounds (Gowda *et al.*, 2007*a*,*b*,*c*; Haider *et al.*, 2009) and are within normal ranges (Allen *et al.*, 1987). In the crystal structure, each molecule is linked to an adjacent one through classical O5—H7···O4 intermolecular hydrogen bonds forming centrosymmetric dimers typical of carboxylic acids, Table 1. These dimers are further connected by N—H···O and C—H···O hydrogen bonds to form an extended network, Fig 2.

### Experimental

To an aqueous solution (10.0 ml) of 4-amino benzoic acid (1.0 g; 7.3 mmoles) maintained at pH 9 with aqueous sodium bicarbonate solution, 4-(acetylamino)benzenesulfonyl chloride (2.21 g, 9.48 mmol) was added. Contents were stirred at room temperature until the complete consumption of the sulfonyl chloride (as indicated by TLC). The pH of the reaction mixture was changed to 1 using hydrochloric acid (1 M) and the precipitate obtained was filtered, washed with water and dried. The resulting solid was recrystallized from methanol to get suitable crystals.

### Refinement

All hydrogen atoms were identified in the difference map. Those bonded to O, C and N were fixed in ideal positions and treated as riding on their parent atoms. The following distances were used: methyl C—H 0.98Å; aromatic C—H 0.95Å; N—H 0.86 Å.

### Crystal data

**Table:**

C_15_H_14_N_2_O_5_S	*Z* = 2
*M**_r_* = 334.34	*F*(000) = 348
Triclinic, *P*1	*D*_x_ = 1.423 Mg m^−^^3^
Hall symbol: -P 1	Mo *K*α radiation, λ = 0.71073 Å
*a* = 7.9829 (3) Å	Cell parameters from 5086 reflections
*b* = 8.4143 (3) Å	θ = 2.6–27.5°
*c* = 12.6554 (5) Å	µ = 0.23 mm^−^^1^
α = 70.888 (2)°	*T* = 296 K
β = 81.553 (2)°	Needles, dark brown
γ = 77.104 (2)°	0.24 × 0.18 × 0.14 mm
*V* = 780.44 (5) Å^3^	

### Data collection

**Table:**

Bruker APEXII CCD area-detector diffractometer	2928 reflections with *I* > 2σ(*I*)
Radiation source: fine-focus sealed tube	*R*_int_ = 0.032
graphite	θ_max_ = 28.3°, θ_min_ = 2.7°
φ and ω scans	*h* = −9→10
13620 measured reflections	*k* = −10→11
3835 independent reflections	*l* = −16→16

### Refinement

**Table:**

Refinement on *F*^2^	Primary atom site location: structure-invariant direct methods
Least-squares matrix: full	Secondary atom site location: difference Fourier map
*R*[*F*^2^ > 2σ(*F*^2^)] = 0.054	Hydrogen site location: inferred from neighbouring sites
*wR*(*F*^2^) = 0.150	H-atom parameters constrained
*S* = 1.02	*w* = 1/[σ^2^(*F*_o_^2^) + (0.0733*P*)^2^ + 0.3675*P*] where *P* = (*F*_o_^2^ + 2*F*_c_^2^)/3
3835 reflections	(Δ/σ)_max_ = 0.034
210 parameters	Δρ_max_ = 0.49 e Å^−^^3^
0 restraints	Δρ_min_ = −0.47 e Å^−^^3^

### Special details

**Table:**

Geometry. All e.s.d.'s (except the e.s.d. in the dihedral angle between two l.s. planes) are estimated using the full covariance matrix. The cell e.s.d.'s are taken into account individually in the estimation of e.s.d.'s in distances, angles and torsion angles; correlations between e.s.d.'s in cell parameters are only used when they are defined by crystal symmetry. An approximate (isotropic) treatment of cell e.s.d.'s is used for estimating e.s.d.'s involving l.s. planes.
Refinement. Refinement of *F*^2^ against ALL reflections. The weighted *R*-factor *wR* and goodness of fit *S* are based on *F*^2^, conventional *R*-factors *R* are based on *F*, with *F* set to zero for negative *F*^2^. The threshold expression of *F*^2^ > σ(*F*^2^) is used only for calculating *R*-factors(gt) *etc*. and is not relevant to the choice of reflections for refinement. *R*-factors based on *F*^2^ are statistically about twice as large as those based on *F*, and *R*- factors based on ALL data will be even larger.

### Fractional atomic coordinates and isotropic or equivalent isotropic displacement parameters (Å^2^)

**Table:**

	*x*	*y*	*z*	*U*_iso_*/*U*_eq_	
S1	0.36529 (6)	0.60917 (7)	0.20813 (4)	0.03843 (18)	
N1	0.1928 (2)	0.5659 (2)	0.28924 (14)	0.0397 (4)	
H1	0.1346	0.4996	0.2775	0.048*	
O2	0.50673 (18)	0.5656 (2)	0.27558 (14)	0.0503 (4)	
O1	0.3732 (2)	0.5277 (2)	0.12378 (13)	0.0489 (4)	
C4	0.2682 (3)	1.1815 (3)	0.03783 (17)	0.0380 (5)	
C1	0.3286 (2)	0.8313 (3)	0.14368 (17)	0.0373 (4)	
N3	0.2462 (2)	1.3587 (2)	−0.01344 (14)	0.0424 (4)	
H3	0.2964	1.4127	0.0158	0.051*	
O16	0.0723 (2)	1.3984 (2)	−0.14922 (15)	0.0601 (5)	
C7	0.1369 (3)	0.6366 (3)	0.37967 (17)	0.0370 (4)	
O5	−0.3233 (3)	0.9184 (3)	0.57458 (17)	0.0786 (7)	
C14	0.1564 (3)	1.4568 (3)	−0.10295 (18)	0.0421 (5)	
O4	−0.3591 (3)	0.9200 (4)	0.40422 (19)	0.0954 (9)	
C9	−0.0886 (3)	0.7910 (3)	0.47573 (18)	0.0437 (5)	
C12	0.2427 (3)	0.6086 (3)	0.46374 (19)	0.0511 (6)	
H12	0.3535	0.5447	0.4610	0.061*	
C8	−0.0291 (3)	0.7287 (3)	0.38531 (18)	0.0411 (5)	
H8	−0.1007	0.7490	0.3288	0.049*	
C10	0.0194 (3)	0.7651 (3)	0.5583 (2)	0.0530 (6)	
H10	−0.0194	0.8083	0.6182	0.064*	
C11	0.1851 (3)	0.6745 (4)	0.5510 (2)	0.0593 (7)	
H11	0.2586	0.6580	0.6058	0.071*	
C15	0.1669 (3)	1.6414 (3)	−0.1392 (2)	0.0518 (6)	
H15A	0.0989	1.7018	−0.2019	0.078*	
H15B	0.2847	1.6541	−0.1608	0.078*	
H15C	0.1237	1.6877	−0.0784	0.078*	
C6	0.2468 (5)	0.9002 (4)	0.0475 (3)	0.0854 (11)	
H6	0.2102	0.8281	0.0172	0.102*	
C5	0.2170 (5)	1.0723 (4)	−0.0058 (3)	0.0823 (11)	
H5	0.1618	1.1157	−0.0720	0.099*	
C13	−0.2682 (3)	0.8827 (4)	0.4850 (2)	0.0570 (7)	
C2	0.3792 (5)	0.9385 (4)	0.1877 (2)	0.0757 (10)	
H2	0.4357	0.8940	0.2533	0.091*	
C3	0.3476 (5)	1.1122 (4)	0.1358 (2)	0.0760 (10)	
H4	0.3807	1.1841	0.1679	0.091*	
H7	−0.4296	0.9779	0.5730	0.30 (4)*	

### Atomic displacement parameters (Å^2^)

**Table:**

	*U*^11^	*U*^22^	*U*^33^	*U*^12^	*U*^13^	*U*^23^
S1	0.0298 (3)	0.0460 (3)	0.0443 (3)	−0.0019 (2)	−0.0027 (2)	−0.0237 (2)
N1	0.0351 (9)	0.0448 (10)	0.0441 (10)	−0.0086 (7)	−0.0023 (7)	−0.0196 (8)
O2	0.0323 (8)	0.0616 (10)	0.0597 (10)	0.0010 (7)	−0.0102 (7)	−0.0257 (8)
O1	0.0472 (9)	0.0552 (10)	0.0543 (9)	−0.0056 (7)	0.0007 (7)	−0.0346 (8)
C4	0.0325 (10)	0.0502 (12)	0.0366 (10)	−0.0100 (9)	−0.0007 (8)	−0.0199 (9)
C1	0.0319 (9)	0.0462 (12)	0.0393 (11)	−0.0091 (8)	0.0007 (8)	−0.0208 (9)
N3	0.0442 (10)	0.0486 (11)	0.0414 (10)	−0.0124 (8)	−0.0087 (8)	−0.0185 (8)
O16	0.0570 (10)	0.0703 (12)	0.0606 (11)	−0.0117 (9)	−0.0257 (8)	−0.0210 (9)
C7	0.0352 (10)	0.0405 (11)	0.0341 (10)	−0.0052 (8)	−0.0019 (8)	−0.0112 (9)
O5	0.0565 (11)	0.1177 (18)	0.0653 (12)	0.0201 (11)	−0.0089 (9)	−0.0554 (12)
C14	0.0339 (10)	0.0570 (13)	0.0385 (11)	−0.0055 (9)	0.0001 (8)	−0.0221 (10)
O4	0.0579 (12)	0.156 (2)	0.0790 (14)	0.0398 (13)	−0.0287 (11)	−0.0740 (15)
C9	0.0404 (11)	0.0497 (13)	0.0409 (12)	−0.0025 (9)	−0.0047 (9)	−0.0169 (10)
C12	0.0372 (11)	0.0699 (16)	0.0423 (12)	0.0038 (11)	−0.0099 (9)	−0.0182 (11)
C8	0.0372 (10)	0.0485 (12)	0.0376 (11)	−0.0019 (9)	−0.0089 (8)	−0.0146 (9)
C10	0.0522 (13)	0.0692 (16)	0.0402 (12)	−0.0033 (12)	−0.0059 (10)	−0.0244 (12)
C11	0.0489 (13)	0.089 (2)	0.0423 (13)	−0.0005 (13)	−0.0159 (10)	−0.0255 (13)
C15	0.0544 (14)	0.0541 (14)	0.0452 (13)	−0.0014 (11)	−0.0027 (10)	−0.0190 (11)
C6	0.130 (3)	0.0517 (16)	0.096 (2)	−0.0114 (17)	−0.075 (2)	−0.0271 (15)
C5	0.129 (3)	0.0534 (16)	0.080 (2)	−0.0076 (17)	−0.071 (2)	−0.0206 (14)
C13	0.0486 (13)	0.0706 (17)	0.0553 (15)	0.0080 (12)	−0.0088 (11)	−0.0347 (13)
C2	0.128 (3)	0.0570 (16)	0.0561 (16)	−0.0268 (17)	−0.0497 (17)	−0.0113 (13)
C3	0.129 (3)	0.0529 (15)	0.0634 (17)	−0.0294 (17)	−0.0507 (18)	−0.0142 (13)

### Geometric parameters (Å, °)

**Table:**

S1—O2	1.4252 (15)	O4—C13	1.255 (3)
S1—O1	1.4331 (15)	C9—C10	1.383 (3)
S1—N1	1.6264 (17)	C9—C8	1.390 (3)
S1—C1	1.753 (2)	C9—C13	1.477 (3)
N1—C7	1.428 (2)	C12—C11	1.369 (3)
N1—H1	0.8600	C12—H12	0.9300
C4—C3	1.368 (3)	C8—H8	0.9300
C4—C5	1.372 (3)	C10—C11	1.378 (4)
C4—N3	1.399 (3)	C10—H10	0.9300
C1—C2	1.359 (3)	C11—H11	0.9300
C1—C6	1.359 (3)	C15—H15A	0.9600
N3—C14	1.357 (3)	C15—H15B	0.9600
N3—H3	0.8600	C15—H15C	0.9600
O16—C14	1.219 (3)	C6—C5	1.365 (4)
C7—C8	1.383 (3)	C6—H6	0.9300
C7—C12	1.383 (3)	C5—H5	0.9300
O5—C13	1.260 (3)	C2—C3	1.374 (4)
O5—H7	0.8831	C2—H2	0.9300
C14—C15	1.487 (3)	C3—H4	0.9300
			
O2—S1—O1	119.27 (9)	C7—C8—C9	119.82 (19)
O2—S1—N1	108.91 (9)	C7—C8—H8	120.1
O1—S1—N1	105.07 (9)	C9—C8—H8	120.1
O2—S1—C1	107.95 (10)	C11—C10—C9	119.4 (2)
O1—S1—C1	108.32 (10)	C11—C10—H10	120.3
N1—S1—C1	106.68 (9)	C9—C10—H10	120.3
C7—N1—S1	120.87 (14)	C12—C11—C10	120.6 (2)
C7—N1—H1	119.6	C12—C11—H11	119.7
S1—N1—H1	119.6	C10—C11—H11	119.7
C3—C4—C5	117.9 (2)	C14—C15—H15A	109.5
C3—C4—N3	118.54 (19)	C14—C15—H15B	109.5
C5—C4—N3	123.5 (2)	H15A—C15—H15B	109.5
C2—C1—C6	118.4 (2)	C14—C15—H15C	109.5
C2—C1—S1	121.67 (18)	H15A—C15—H15C	109.5
C6—C1—S1	119.95 (17)	H15B—C15—H15C	109.5
C14—N3—C4	128.83 (18)	C1—C6—C5	121.8 (2)
C14—N3—H3	115.6	C1—C6—H6	119.1
C4—N3—H3	115.6	C5—C6—H6	119.1
C8—C7—C12	119.55 (19)	C6—C5—C4	120.2 (2)
C8—C7—N1	119.05 (18)	C6—C5—H5	119.9
C12—C7—N1	121.34 (19)	C4—C5—H5	119.9
C13—O5—H7	113.2	O4—C13—O5	123.1 (2)
O16—C14—N3	122.6 (2)	O4—C13—C9	118.9 (2)
O16—C14—C15	122.7 (2)	O5—C13—C9	118.0 (2)
N3—C14—C15	114.73 (19)	C1—C2—C3	120.4 (2)
C10—C9—C8	120.1 (2)	C1—C2—H2	119.8
C10—C9—C13	119.8 (2)	C3—C2—H2	119.8
C8—C9—C13	120.1 (2)	C4—C3—C2	121.2 (2)
C11—C12—C7	120.4 (2)	C4—C3—H4	119.4
C11—C12—H12	119.8	C2—C3—H4	119.4
C7—C12—H12	119.8		
			
O2—S1—N1—C7	55.28 (18)	C13—C9—C8—C7	−177.2 (2)
O1—S1—N1—C7	−175.87 (15)	C8—C9—C10—C11	−1.1 (4)
C1—S1—N1—C7	−60.99 (17)	C13—C9—C10—C11	177.9 (3)
O2—S1—C1—C2	−23.6 (3)	C7—C12—C11—C10	2.0 (4)
O1—S1—C1—C2	−154.0 (2)	C9—C10—C11—C12	−0.8 (4)
N1—S1—C1—C2	93.4 (2)	C2—C1—C6—C5	0.8 (5)
O2—S1—C1—C6	156.7 (2)	S1—C1—C6—C5	−179.4 (3)
O1—S1—C1—C6	26.3 (3)	C1—C6—C5—C4	−0.7 (6)
N1—S1—C1—C6	−86.4 (3)	C3—C4—C5—C6	−0.6 (5)
C3—C4—N3—C14	−172.3 (3)	N3—C4—C5—C6	178.3 (3)
C5—C4—N3—C14	8.9 (4)	C10—C9—C13—O4	173.0 (3)
S1—N1—C7—C8	124.75 (19)	C8—C9—C13—O4	−8.0 (4)
S1—N1—C7—C12	−57.9 (3)	C10—C9—C13—O5	−7.6 (4)
C4—N3—C14—O16	3.6 (3)	C8—C9—C13—O5	171.4 (3)
C4—N3—C14—C15	−177.18 (19)	C6—C1—C2—C3	0.2 (5)
C8—C7—C12—C11	−1.3 (4)	S1—C1—C2—C3	−179.5 (3)
N1—C7—C12—C11	−178.6 (2)	C5—C4—C3—C2	1.6 (5)
C12—C7—C8—C9	−0.6 (3)	N3—C4—C3—C2	−177.3 (3)
N1—C7—C8—C9	176.8 (2)	C1—C2—C3—C4	−1.5 (5)
C10—C9—C8—C7	1.8 (4)		

### Hydrogen-bond geometry (Å, °)

**Table:**

*D*—H···*A*	*D*—H	H···*A*	*D*···*A*	*D*—H···*A*
O5—H7···O4^i^	0.88	1.74	2.617 (4)	170
N1—H1···O16^ii^	0.86	2.31	2.860 (2)	122
N3—H3···O1^iii^	0.86	2.13	2.974 (2)	165
C11—H11···O2^iv^	0.93	2.59	3.379 (3)	143

Symmetry codes: (i) −*x*−1, −*y*+2, −*z*+1; (ii) −*x*, −*y*+2, −*z*; (iii) *x*, *y*+1, *z*; (iv) −*x*+1, −*y*+1, −*z*+1.
